# Phase II trial of biweekly docetaxel, cisplatin, and 5-fluorouracil chemotherapy for advanced esophageal squamous cell carcinoma

**DOI:** 10.1007/s00280-016-2985-y

**Published:** 2016-02-20

**Authors:** Yoshihiro Tanaka, Kazuhiro Yoshida, Atsuko Yamada, Toshiyuki Tanahashi, Naoki Okumura, Nobuhisa Matsuhashi, Kazuya Yamaguchi, Tatsuhiko Miyazaki

**Affiliations:** Department of Surgical Oncology, Gifu Graduate School of Medicine, 1-1 Yanagido, Gifu, 501-1194 Japan; Department of Pathology, Gifu University Hospital, Gifu, Japan

**Keywords:** Chemotherapy, Docetaxel, Cisplatin, 5-fluorouracil, Esophageal carcinoma

## Abstract

The prognosis of esophageal cancer patients is still unsatisfactory. Although a docetaxel, cisplatin, and 5-Fu (DCF) regimen has been reported, it is often difficult to accomplish because of severe toxicity. Therefore, we developed a new biweekly DCF (Bi-DCF) regimen and previously reported the recommended dose in a phase I dose-escalation study. We then performed a phase II study of Bi-DCF for advanced esophageal squamous cell carcinoma (SCC). Patients with clinical stage II/III were eligible. Patients received 2 courses of chemotherapy: docetaxel 35 mg/m^2^ with cisplatin 40 mg/m^2^ on days 1 and 15 and 400 mg/m^2^ 5-fluorouracil on days 1–5 and 15–19 every 4 weeks. After completion of the chemotherapy, patients received esophagectomy. The primary endpoint was the completion rate of protocol treatment. Thirty-two patients were enrolled. The completion rate of protocol treatment (completion of two courses of preoperative chemotherapy and R0 surgery) was 100 %. During chemotherapy, the most common grade 3 or 4 toxicities were neutropenia (31.3 %). No treatment-related death was observed, and the incidence of operative morbidity was tolerable. The overall response rate after the chemotherapy was 90.3 %. This Bi-DCF regimen was well tolerated and highly active. This trial was registered with the University Hospital Medical Information Network (No. UMIN 000014625).

## Introduction

Despite improvements in the modalities for early diagnosis of esophageal cancer, the majority of patients still tend to have widespread disease at the time of diagnosis [1]. Locally advanced esophageal carcinomas are treated with current modalities for early diagnosis and perioperative multimodalities. Although morbidity and mortality rates after surgical treatment for advanced esophageal cancer have decreased, the 5-year survival rate after curative surgery is still only 20–36 % [2]. In patients with operable esophageal cancer, there is evidence supporting the use of preoperative chemotherapy [3, 4], but optimal chemotherapy for this disease has not been defined.

Patients with obvious T4 (non-resectable) or inoperable disease are usually treated with various chemotherapy strategies or chemoradiotherapy. However, their survival is still not satisfactory [5–8]. The most frequently used chemotherapy regimen for patients with metastatic disease is a combination of 5-fluorouracil (5-Fu) and cisplatin (CDDP), with response rates ranging from 15 to 45 % [9].

Meanwhile, many chemotherapy regimens for locally advanced tumors have been reported to date. Despite the availability of various chemotherapy regimens, advanced esophageal cancer carries a very poor prognosis, with a mean survival time of less than 8.1 months with current chemotherapies used singly or in combination with 5-FU, vindesine, mitomycin, docetaxel (TXT), paclitaxel, CDDP, irinotecan, vinorelbine, or capecitabine [10]. Fluorouracil and CDDP combination therapy (FP) is regarded as standard [11], for which the median survival time is reported to be 9.2 months for responders and 5.3 months for non-responders [12]. The response rates reported with FP range from 35 to 40 %, whereas the 2-year survival rates of patients with locally advanced esophageal cancer range from 8 to 55 %, with a mean of 27 % [13–15].

The next generation of regimens to treat both distant metastasis and locally advanced cancer has begun to be developed. Many studies have shown that the taxanes have significant activity in patients with locally advanced and metastatic esophageal carcinomas [16–20].

The combination of TXT, CDDP, and 5-FU (DCF) has shown activity in upper gastrointestinal malignancies with different mechanisms. In Europe, DCF combination therapy is commonly used in patients with advanced gastric cancer. In a report from the V325 study group, patients with advanced gastric or gastroesophageal junction cancer receiving DCF not only had statistically improved overall survival and time-to-tumor progression, but they also had better preservation of quality of life compared with patients receiving FP therapy [21]. Advanced esophageal cancer might also benefit from the possibility of such a triple-combination regimen.

It is now necessary to reduce both the hematological and non-hematological toxicities as much as possible. Chemotherapy can significantly improve the clinical outcomes of cancer patients, but it can also cause serious adverse effects [22]. Treatment with DCF in esophageal cancer is reported to be associated with increased response rates but also with a highly increased incidence of toxicities, the most common of which are hematological and gastrointestinal [23, 24]. Thus, there is an urgent need for the creation of more effective treatment regimens with fewer adverse events. We therefore previously conducted a phase I clinical trial of DCF in patients with advanced thoracic esophageal squamous cell carcinoma (SCC) with T3–4 tumors and/or M1 staging with divided administration of each drug into two blocks per one course, i.e., biweekly DCF (Bi-DCF). We determined the recommended dose for use in phase II trials to be TXT 35 mg/m^2^, CDDP 40 mg/m^2^, and 5-Fu 400 mg/m, as was already reported [22]. In particular, preoperative chemotherapy for advanced esophageal carcinoma requires tolerability, because subsequent radical surgery for esophageal cancer is invasive for patients. We then carried out a phase II single-center study of preoperative chemotherapy with Bi-DCF in patients with clinical stage II/III thoracic esophageal SCC.

## Patients and methods

### Patient eligibility criteria

To be eligible for the study, patients had to be at least 18 years of age at the time of enrollment and have histologically or cytologically confirmed SCC, which was a locally advanced clinical stage II/III (International Union Against Cancer TNM classification system, 7th edition [25]). They also had to have an Eastern Cooperative Oncology Group (ECOG) performance status of 0–1, a life expectancy of >12 weeks, and adequate liver, bone marrow, renal, and cardiovascular function as indicated by a serum bilirubin ≤1.5 mg/dl, neutrophil count ≥1500/mm^3^, serum aspartate aminotransferase and alanine aminotransferase ≤twice the upper limit of normal range, platelet count ≥10 × 10^4^/mm^3^, hemoglobin ≥8.0 g/dl, and creatinine ≤1.2 mg/dl (or creatinine clearance >60 ml/min). Patients previously treated with chemotherapy for disease or irradiation to major bone areas were excluded from the study. The major exclusion criteria included serious concomitant illness, symptomatic infectious disease, severe drug allergy, symptomatic peripheral neuropathy, or uncontrolled diabetes mellitus.

### Study approval

All participants had to sign an informed consent form approved by the Ethical Committee of Gifu University Hospital before study entry.

### Treatment plan

#### Chemotherapy

Patients received TXT diluted in 250 ml of normal saline at a dose of 35 mg/m^2^, which was infused intravenously (iv) over 2 h, followed by a maintenance infusion given for the next 2 h. Then, CDDP was prepared in normal saline at a dose of 40 mg/m^2^ and administered iv over 2 h on day 1. 5-FU was prepared in normal saline at a dose of 400 mg/m^2^ and administered iv continuously on days 1–5. TXT and CDDP were given on days 1 and 15, and 5-FU was given on days 1–5 and 15–19 of every 28-day cycle (one course). All patients were premedicated with granisetron 2 mg iv. Hypersensitivity reactions were treated with prophylactic use of dexamethasone 8 mg iv, which was infused 1 h prior to the administration of TXT. Further, dexamethasone was prescribed at a dose of 4 mg orally for 2 days after administration of the TXT to reduce the risk of hypersensitivity reaction and fluid retention. Diuretics were added at the discretion of the treating physician. Appropriate hydration was given before and after the CDDP infusion. Antiemetics were recommended on subsequent days as needed. The protocol did not allow the use of prophylactic granulocyte colony-stimulating factor (G-CSF) and antimicrobial therapy during chemotherapy. Patients were treated with hospitalization at first course. As long as serious adverse events did not occurred, all patients discharged at second course except days of drug administration.

### Treatment assessment and dose modifications

All patients underwent complete staging procedures to document disease extent, including ECOG performance status, medical history, and physical examination. Clinicopathological factors were analyzed based on the TNM classification [25].

Prestudy laboratory evaluation, including a complete blood cell count, serum electrolytes, urea, creatinine and 24-h creatinine clearance, bilirubin, alkaline phosphatase and transaminases, CEA, SCC, CA19-9, and CYFRA measurements, and electrocardiogram were obtained within 1 week before initiation of treatment and at the start of each treatment cycle. Baseline computed tomography (CT) or magnetic resonance imaging (MRI) scans were performed within 4 weeks prior to study entry. All patients had a complete blood count taken every week during chemotherapy. Levels of electrolytes, serum creatinine, transaminases, alkaline phosphatase and bilirubin, and plasma urea were measured every week until receiving surgery.

Toxicity was graded every week during the study according to the US National Cancer Institute—Common Terminology Criteria for Adverse Events (NCI-CTC) version 4.0 [26].

Measurable lesions except for the primary tumor were evaluated by CT or MRI and assessed according to the Response Evaluation Criteria in Solid Tumors (RECIST) [27]. A complete response was defined as complete disappearance of all clinically detectable malignant disease. A partial response was defined as a ≥30 % decrease in the sum of the perpendicular diameters of all measurable lesions lasting at least 4 weeks. Progressive disease was defined as a ≥20 % increase in the sum of the products of measurable lesions over the smallest sum observed, or the appearance of new lesions. Stable disease did not qualify as complete response, partial response, or progressive disease. Close follow-up was made from both endoscopy and radiographic films or scans taken to document treatment response during therapy and was repeated in the 4th week of every course of treatment and 4 weeks after completion of the 2 courses or sooner if the patient appeared to show disease progression.

Response rate (complete or partial) was confirmed 4 weeks after completion of the 2 courses of chemotherapy. Then, patients received right thoracotomy with thoracic esophagectomy within 14 days. A thoracoscopic surgery was permitted. If disease progression or new metastasis was detected after 1 course, the subsequent cycle was not permitted, and immediate surgery or chemoradiation was mandated. Regional lymphadenectomy consisted of two- or three-field extended lymphadenectomy. Evaluations of residual tumor (R) were classified as follows: R0, no residual tumor; R1, suspicious of residual tumor or microscopic residual tumor; or R2, macroscopic residual tumor.

After surgery, the primary tumor was examined for histopathological changes using a grading system by the Japanese Classification of Esophageal Carcinoma [28], with the following grades: grade 3 (markedly effective; no viable cancer cells; pathologically complete response), grade 2 (moderately effective; viable cancer cells account for less than 1/3 of tumor tissue, whereas other cancer cells show severe degeneration or necrosis), and grade 1 (slightly effective, where apparently viable cancer cells account for 1/3 or more of the tumor tissue, but there is some evidence of degeneration of cancer tissue or cells). Grade 1 lesions were also subclassified into grade 1a (viable cancer cells account for ≥2/3 of tumor tissue), grade 1b (viable cancer cells account for ≥1/3, but <2/3, of tumor tissue), and grade 0 (ineffective, denoting no discernible therapeutic effect on cancer tissue or cells).

Dose adjustment was carried out by reducing the doses of TXT, CDDP, and 5-Fu by 20 % in the subsequent course if grade 4 hematological toxicity or grade 3 or 4 non-hematological toxicity was present. If there was no improvement in grade 3/4 toxicity on the day of predetermined course, we postponed the chemotherapy.

We terminated the protocol treatment if serious adverse reactions were manifested, clear progression of the disease was observed, or the physician otherwise judged that administration should be stopped.

### Endpoints and statistical methods

The primary objectives of this phase II study was the compliance with treatment completion. Patients were considered to have completed treatment if they received 2 courses of chemotherapy and pathologically proven complete resection (R0). Secondary objectives included: the safety and tolerability of this chemotherapy; evaluation of operative morbidity and mortality; and evaluation of efficacy including response rate, pathological response, 1-year relapse-free survival (RFS), and overall survival (OS).

In this phase II trial, we expected that the clinical incidence of toxicities with Bi-DCF would increase above that with FP in the preoperative design, and that the rate of treatment completion would be lower than that in JCOG 9907 (89.6 %) [29]. Accordingly, we assumed a null hypothesis with a 75 % completion rate for protocol treatment and expected a completion rate of protocol treatment of 90 %. Given a one-sided alpha of 0.1 and statistical power of 80 %, a minimum of 28 patients was needed. Assuming a dropout rate of 10 %, the projected sample size was 32 patients in total.

Relapse-free survival was defined as the time from the date of registration to the first documentation of disease recurrence. Overall survival was measured from the date of registration to the date of the last follow-up or death. Statistical data were obtained using the SPSS 20.0 software package (SPSS Inc., Chicago, IL, USA).

This trial was registered with the University Hospital Medical Information Network (No. UMIN 000014625).

## Results

### Patient characteristics

Between January 2010 and September 2014, 32 patients were enrolled into the study. Demographic and clinical characteristics of the study population are summarized in Table 1. All patients had locally advanced esophageal SCC. The median patient age was 68 years (range 40–75 years). All patients had an ECOG performance status of 0–1. Finally, 32 patients were enrolled, and all patients fully underwent Bi-DCF therapy.

**Table 1:** Characteristics of patients

Characteristics	No. of patients (*N* = 32)	%
Age, years		
Median	68	
Range	40–75	
Sex		
Males	26	81.3
Females	6	18.8
ECOG performance status		
0–1	32	100
2	0	0
Site of primary tumor		
Ut	8	25.0
Mt	15	46.9
Lt	9	28.1
Clinical T stage		
cT2	8	25.0
cT3	24	75.0
Clinical *N* stage		
cN0	1	3.1
cN1	9	28.1
cN2	13	40.6
cN3	9	28.1
Clinical stage		
IIB	3	9.4
III	29	90.6

*ECOG* Eastern Cooperative Oncology Group, *Ut* upper thoracic esophagus, *Mt* middle thoracic esophagus, *Lt* lower thoracic esophagus

### Toxicity

Overall toxicities during chemotherapy are listed in Table 2. The major toxicities were leukopenia and neutropenia. Two patients (6.3 %) had grade 4 and 8 patients (25 %) had grade 3 neutropenia; however, no patients had febrile neutropenia. Two patient with grade 4 neutropenia received G-CSF. Common non-hematological adverse events were anorexia, fatigue, mucositis, diarrhea, and alopecia. No grade 3 or 4 hyponatremia was occurred. All events were below grade 2. No treatment-related deaths occurred. All toxicities were within expectations and were manageable.

**Table 2:** Frequency of treatment-related toxicity

	CTCAE version 4.0 common toxicity criteria					
	Grade 1	Grade 2	Grade 3	Grade 4	All grades (%)	Grade 3/4 (%)
Hematological						
Leucopenia	5	12	4	0	21 (65.6)	4 (12.5)
Neutropenia	2	7	8	2	19 (59.4)	10 (31.3)
Febrile neutropenia	–	–	0	0	0	0
Anemia	0	0	0	0	0	0
Thrombocytopenia	0	1	0	0	1 (3.1)	0
Non-hematological						
Anorexia	8	5	0	0	13 (40.6)	0
Fatigue	2	1	0	0	3 (9.4)	0
Mucositis	2	9	0	0	11 (34.4)	0
Nausea/vomiting	1	0	0	0	1 (3.1)	0
Diarrhea	2	3	0	0	5 (15.6)	0
Pericardial effusion	–	0	0	0	0	0
Alopecia	23	9	–	–	32 (100)	0
Edema	5	0	0	0	5 (15.6)	0
Sensory neuropathy	0	0	0	0	0	0
Dysgeusia	1	0	0	0	1 (3.1)	0
Hyponatremia	–	–	0	0	0	0

Data represent number of patients

*CTCAE* Common Terminology Criteria for Adverse Events of the National Cancer Institute

### Surgery and postoperative complications

All patients received surgery as listed in Table 3. Subtotal esophagectomy via right thoracotomy with two- or three-field lymphadenectomy was performed in 30 patients. Two patients received subtotal esophagectomy and lymphadenectomy via thoracoscopic surgery. We undergone reconstruction by stomach roll using subtotal stomach and hand-sewn anastomosis in cervical portion in all cases. All patients were considered to have achieved curative resection (R0).

**Table 3:** Operative details and postoperative outcomes

	No of patients	%
Surgical approach		
Right thoracotomy	30	93.8
Thoracoscopic surgery	2	6.3
Type of resection		
R0	32	100
R1	0	0
Postoperative complications		
Recurrent nerve palsy	3	9.4
Pneumonia	0	0
Anastomotic leakages	0	0
Pyothorax	0	0
Pneumothorax	0	0
Chylothorax	1	3.1
Wound infection	0	0
Heart failure	0	0
Postoperative mortality	0	0

*R0* no residual tumor, *R1* suspicious of residual tumor or microscopic residual tumor

Of the 32 patients who received surgery, postoperative complications (grade 2 or more according to NCI-CTCAE version 4.0) occurred in 3 patients in the form of grade 2 recurrent nerve palsy and in 1 patient in the form of grade 2 chylothorax. There were no anastomotic leakage and postoperative deaths.

### Treatment outcomes

Of all 32 patients, no patients failed to complete 2 courses of chemotherapy and no patients required a delay of chemotherapy during any of the courses for adverse events. No patients required a dose reduction in the all courses because grade 4 neutropenia occurred after the completion of the 2 courses of the regimen.

All the patients received the surgery, and no patients required R1 resection pathologically. Thus, the completion rate (completion of 2 courses of preoperative chemotherapy and R0 surgery) of protocol treatment was 100 %.

Of the 31 patients who had measurable lesions, 6 (19.4 %) had a complete response and 22 (71.0 %) had a partial response to therapy, resulting in an overall response rate of 90.3 % (95 % confidence interval 74.3–98.0 %) (Table 4).

**Table 4:** Overall response in this phase II trial

	*N* = 31
Complete response	6 (19.4 %)
Partial response	22 (71.0 %)
Stable disease	3 (9.7 %)
Progressive disease	0
Overall response rate	90.3 %
Confidence interval	74.3–98.0

The histological effects in the primary tumors were grade 3 in 7 (21.9 %) patients, grade 2 in 10 (31.3 %) patients, grade 1b in 3 (9.4 %) patients, and grade 1a in 12 (37.5 %) patients. The results also showed that 13 (41.9 %) of the 31 patients who were clinically diagnosed as positive for lymph node metastasis were pathologically node negative.

The median follow-up period was 30 months (range 12–68 months). The 1-year RFS was 84.3 %. The 1-year survival rate was 90.6 %. The median RFS was not reached.

## Discussion

Currently, additional treatments following surgery or radiotherapy are necessary to improve long-term patient outcome. In Western countries, neoadjuvant chemoradiotherapy is a standard treatment for locally advanced esophageal cancer [10]. Meanwhile, in Japan, neoadjuvant chemotherapy with FP has been approved as a standard regimen by the JCOG 9907 study [3]. There seemed to be a histological difference in the background of esophageal cancer, and although FP was an effective regimen, the response rate of 38 % was unsatisfactory. Thus, a new regimen with low toxicity and high response is clearly desired.

In the present trial of Bi-DCF chemotherapy for esophageal SCC, a combination regimen with DCF was shown to be highly tolerable and effective in patients with clinical stage II/III cancer in preoperative setting.

The taxanes enhance polymerization of tubulin into stable microtubule formations and inhibit their tubulin depolymerization by blocking the cell cycle in metaphase, anaphase, and interphase [30]. This inhibition may improve the efficacy of drugs such as CDDP, which are active in all phases of the cell cycle via direct DNA damage. Furthermore, the taxanes increase programmed cell death, and TXT appears to be more potent than paclitaxel in inhibiting angiogenesis [31].

TXT, CDDP, and 5-FU activity occurs by synergistic or non-cross-resistance effects when administered in combination [24]. Previously published studies have shown that the DCF combination has good efficacy [23, 32–38]. A 34.5–83.3 % response rate was observed with DCF combinations used to treat patients with advanced esophageal carcinoma (Table 5).

**Table 5:** Docetaxel, CDDP, and 5-Fu for advanced esophageal cancer

References (first author)	Target	Regimen (/m^2^)	Phase	Cases,* n*	Grade 3/4 leukopenia (%)	Grade 3/4 neutropenia (%)	Febrile neutropenia (%)	Response rate (%)	Histopathological response rate (>grade 2) (%)	Histopathological complete response rate (grade 3) (%)	Dose reduction rate in the second cycle (%)	Protocol completion rate (%)
Takahashi [32]	Esophageal cancer (SCC) Stages III, IV	D: 50 (day 1) C: 70 (day 1) F: 700 (days 1–5)/3 wks	I/II	39	53.8	43.6	12.8	66.6	–	–	–	–
Osaka [33]	Esophageal cancer (SCC) Stages III, IV	D: 60 (day 1) C: 60 (day 1) F: 800 (days 1–5)/3–4 wks × 2 courses	II	30	33.3	–	–	83.3 (primary lesion)	–	–	–	96.7
Yamasaki [23]	Esophageal cancer (SCC) Stages III, IV	D: 70 (day 1) C: 70 (day 1) F: 700 (days 1–5)/3 wks × 2 courses	I/II	9/40	72.5	90	10	72.5	40	25	–	82.5
Tamura [34]	Esophageal cancer (SCC) Stage IV	D: 60 (day 1) C: 70 (day 1) F: 600 (days 1–5)/4 wks × 2 courses	II	29	52	76	21	34.5 (confirmed cases)	–	–	13.8	–
Ferri [35]	Esophageal cancer and gastric cancer (AD) Stages II, III, IV	D: 75 (day 1) C: 75 (day 1) F: 750 (days 1–5)/3 wks × 3 courses	II	43	–	20	2.3	–	–	9.8	–	95
Hara [36]	Esophageal cancer (SCC) Stages IIA, IIB, III	D: 70 (day 1) C: 70 (day 1) F: 750 (days 1–5) D: 75 (day 1) C: 75 (day 1) F: 750 (days 1–5)/3 wks × 3 courses	II	42	45.2	83.3	2.4	64.3	51	17	64.3	95.2
Watanabe [37]	Esophageal cancer (SCC, AD) Stages IIB, III, IVA, IVB (TNM6)	D: 60 (day 1) C:6 (days 1–5) F: 350 (days 1–5)/3 wks × 2 courses	Prospective intension-to-treat	50	–	78.2	14.5	53.7	26	12	–	–
Hironaka [38]	Esophageal cancer (SCC, AS, B) Stage IV	D: 30 (days 1, 15) C: 80 (day 1) F: 800 (days 1–5)/4 wks	I/II	10/52	9.1	25.5	0	62	–	–	–	–
Current study	Esophageal cancer (SCC) Stages II, III	D: 35 (days 1, 15) C:40 (days 1, 15) F: 400 (days 1–5, 15–19)	II	32	12.5	31.3	0	90.3	53.2	21.9	0	100

*SCC* squamous cell carcinoma, *AD* adenocarcinoma, *AS* adenosquamous carcinoma, *B* basaloid carcinoma, *D* docetaxel, *C* cisplatin, *F* fluorouracil

We found that our Bi-DCF regimen also had a high response rate and showed highly promising antitumor activity. The histological grade 2/3 rate was 53.2 %, which is high compared with the 25–51 % reported previously [23, 36, 37]. The results emerging from this phase II study are particularly encouraging. By dividing the single dose of each of these three powerful drugs, it is not necessary to reduce their doses in the second course. Because this may eventually increase the dose intensity of the antitumor agent, it is likely that a high response rate can be obtained.

Previous studies indicated that this triplet regimen seems to be not inferior to chemoradiotherapy with respect to the local control rate [23, 39–41]. In the present study, 24 (75.0 %) patients were clinically T3; remarkably, the histological effects regarding T3 tumor were grade 3 in 7 (29.2 %), grade 2 in 8 (33.3 %), grade Ib in 1 (4.2 %), and grade Ia in 8 (33.3 %)patients. Therefore, this regimen might lead to more cases of locally highly advanced esophageal SCC.

Although TXT offers favorable outcomes, it causes adverse hematological toxicity. Because of high rate of blood toxicity resulting from the administration method used in previous reports, there appeared to be difficulties in enforcing the regimen in general hospitals. Neutropenia occurs approximately 8–10 days after TXT administration but recovers rapidly. TXT 75–100 mg/m^2^ every 3–4 weeks is associated with a quite pronounced neutropenia, up to 44 % rate of febrile neutropenia in patients with recurrent ovarian cancer [42]. Several reports revealed that the major toxicity of DCF repeated every 3–4 weeks at doses of TXT 50–75 mg/m^2^, CDDP60-75 mg/m^2^, and 5-FU 700–800 mg/m^2^ was myelosuppression, and the frequencies of grade 3/4 leucopenia and neutropenia in a phase II study were 33.3 % and 90 %, respectively [23, 32–36], (Table 5). In our biweekly regimen, the incidence of hematological toxicity was lower than that in the other reports. Although we did not use a prophylactic antimicrobial agent, no febrile neutropenia occurred, and no thrombocytopenia ≥grade 3 was observed.

To minimize toxicity and maximize the total dose intensity, we elected to investigate a biweekly regimen. Compared with the other DCF regimens for esophageal cancer of other phase I/II studies [23, 32–38], (Table 5), our regimen included a TXT dose intensity equivalent to the triweekly administration of TXT performed in those studies. Divided administration of TXT and CDDP may reduce myelosuppression and neuropathy while maintaining almost unchanged efficacy. Tebbutt et al. [24] showed that weekly TXT administration in the triplet DCF regimen reduced blood toxicity for patients with esophagogastric cancer.

In the present phase II trial, the incidence of TXT-specific toxicities, such as neurotoxicity and acute hypersensitivity reactions [22], was relatively low and did not appear to be a major clinical problem, so a reduction in dose generally was not required. This was probably due to the low per-day dose of TXT administered via the biweekly method. Fluid retention manifesting as peripheral edema, pleural effusion, or ascites was cumulative in incidence and severity, but no patient had severe body weight gain that required diuretics. Patients receiving more than 50 mg/m^2^ of CDDP may suffer nausea and vomiting [43]. Both are frequent side effects and can be well controlled by biweekly method and the administration of granisetron and dexamethasone. Thus, non-hematological toxicity was comparable to that of other reports.

The Bi-DCF regimen seemed to be useful as preoperative chemotherapy due to the small need to delay the anticancer drug administration schedule, and it did not adversely affect our elective surgery schedule. In fact, surgery was carried out according to plan in all 32 patients, and no surgeries were delayed. Moreover, there was no mortality, and no serious postoperative complications occurred.

In conclusion, the preoperative Bi-DCF regimen was well tolerated and useful for the treatment of resectable esophageal SCC. It could potentially be offered as a candidate component of standard regimens for treating resectable esophageal SCC. A further phase III clinical trial using this triplet combination should be pursued in the treatment of advanced esophageal SCC.
